# Association between Serum Alkaline Phosphatase Level and Cerebral Small Vessel Disease

**DOI:** 10.1371/journal.pone.0143355

**Published:** 2015-11-18

**Authors:** Han-Bin Lee, Jinkwon Kim, Sang-Heum Kim, Soonhag Kim, Ok-Joon Kim, Seung-Hun Oh

**Affiliations:** 1 Department of Neurology, CHA Bundang Medical Center, CHA University, Seongnam, South Korea; 2 Department of Radiology, CHA Bundang Medical Center, CHA University, Seongnam, South Korea; 3 Institute for Bio-Medical Convergence, College of Medicine, Catholic Kwandong University, Gangneung-si, South Korea; 4 Catholic Kwandong University International St. Mary’s Hospital, Incheon Metropolitan City, South Korea; The University of Tokyo, JAPAN

## Abstract

**Background:**

Serum alkaline phosphatase (ALP) is a marker of vascular calcification. A high serum ALP level is associated with an increase in cardiovascular events, and predicts poor functional outcome in patients with stroke. We investigated whether serum ALP was associated with cerebral small vessel disease (cSVD) and large cerebral artery stenosis (LCAS).

**Methods:**

We evaluated vascular risk factors, brain magnetic resonance images (MRIs), and MR angiograms from 1,011 neurologically healthy participants. The presence of silent lacunar infarction (SLI) and moderate-to-severe cerebral white matter hyperintensities (MS-cWMH) were evaluated as indices of cSVD on brain MRIs. Findings of extracranial arterial stenosis (ECAS) or intracranial arterial stenosis (ICAS) were considered to be indices of LCAS on MR angiograms.

**Results:**

Subjects with SLI (odds ratio [OR]: 2.09; 95% confidence interval [CI]: 1.27–3.42; *p* = 0.004) and MS-cWMH (OR: 1.48; 95% CI; 1.03–2.13, *p* = 0.036) were significantly more likely to have ALP levels in the third tertile (ALP ≥ 195 IU/L) than the first tertile (ALP ≤ 155 IU/L), after adjusting for cardiovascular risk factors. The mean serum ALP level was significantly higher in patients with SLI or MS-cWMH compared to patients without those findings. After adjustment for confounding factors, the multivariate model found that the statistical significance of serum ALP remained when the presence of SLI (OR: 1.05 per 10 IU/L increase in ALP; 95% CI: 1.02–1.08; *p* = 0.003) or MS-cWMH (OR: 1.03 per 10 IU/L increase in ALP; 95% CI: 1.00–1.06; *p* = 0.025) were added to the model. There were no differences in the proportions of patients with LCAS, ICAS, and ECAS across the serum ALP tertiles.

**Conclusions:**

Our study of neurologically healthy participants found a positive association between serum ALP level and indicators of cSVD, but no association between serum ALP level and the indicators of LCAS.

## Introduction

Alkaline phosphatase (ALP) testing is a routine method for evaluating liver and bone disease. Because ALP promotes vascular calcification by hydrolyzing and thereby inactivating organic pyrophosphate [1], it is thought to be a marker of atherosclerosis. Several clinical studies have shown that an increased serum ALP level is an independent predictor of future cardiovascular events [2–5], and another study demonstrated that a high serum ALP level is associated with poor functional outcome and increased risk of mortality in patients with ischemic stroke [6, 7]. The findings of these studies suggest that an increased serum ALP level may have a pathophysiological role in the development of atherosclerotic vascular disease of the heart and brain.

Cerebral small vessel disease (cSVD) is a cerebrovascular disease that involves the occlusion of small perforating arteries or arterioles. Silent brain infarction (SBI) and cerebral white matter hyperintensities (cWMH) are radiological markers of cSVD that are also occasionally found in healthy people. Although both conditions are clinically asymptomatic, they are associated with an increased risk of future stroke [8] and dementia [9]. Distinct from large artery atherosclerosis (eg, carotid artery or middle cerebral artery), they are associated with different pathologic findings, including lipohyalinosis and microatheroma [10].

Emerging evidence suggests a close relationship between cSVD and arterial calcification [11, 12]. Furthermore, cSVD is more frequently observed in patients with elevated serum ALP levels than in patients with lower levels [13]. These findings suggest that vascular calcification is involved in the development of cerebral microangiopathy, and/or that ALP may be a marker for cSVD as well as large cerebral artery atherosclerosis. However, the association between serum ALP and cSVD has yet to be established and should be validated by an independent cohort study. Furthermore, whether or not serum ALP levels have a greater impact on small or large cerebral arteries is unclear, because most of the previous studies focused on only one condition, cSVD or large cerebral artery atherosclerosis, and did not evaluate both conditions simultaneously. Here, we investigated the relationship between serum ALP level and indicators of cSVD and large cerebral artery stenosis (LCAS) in neurologically healthy study participants.

## Methods

### Study design

This was a hospital-based, cross-sectional study. The study participants were individuals without stroke, aged ≥ 45 years, who visited the outpatient clinic of the Department of Neurology or healthcare center at CHA Bundang Medical Center, Seoul, Korea for routine health examinations between March 2008 and December 2014. All of the participants were neurologically healthy, but presented for medical attention because they had underlying cardiovascular risk factors or a family history of stroke. We only included individuals who underwent brain magnetic resonance imaging (MRI) and magnetic resonance angiography (MRA). We reviewed the medical records, results of laboratory testing, and radiological findings of all the study participants, which were extracted from a database. We included only patients whose records contained adequate demographic, radiological, and laboratory data. Of 1,441 study patients extracted from our database during the study period, we excluded 430 for the following reasons: (1) inadequate medical information (n = 93); (2) no laboratory tests performed (n = 154); (3) no data on brain MRI or MRA (n = 84); (4) previous history of neurological disease (n = 39); (5) abnormal neurological findings at the time of examination (n = 28); and (6) history of liver disease, including active hepatitis, liver cirrhosis, and hepatoma (n = 32). A total of 1011 patients were included in this study. Each patient’s data was de-identified prior to analysis. The Institutional Review Board (IRB) of CHA Bundang Medical Center approved the study (IRB no.: BD-2010-083).

### Evaluated clinical characteristics

Patient data included the following: gender, hypertension, diabetes mellitus (DM), current smoking, hypercholesterolemia, and coronary artery occlusive disease (CAOD). Hypertension was diagnosed in patients with systolic blood pressures ≥ 140 mm Hg or diastolic blood pressures ≥ 90 mmHg on repeated measurement, or the patient was on antihypertensive medication. DM was diagnosed if the patient had a fasting plasma glucose ≥ 126 mg/dL or was taking antidiabetic medications or insulin. Hypercholesterolemia was diagnosed if the patient had a total cholesterol ≥ 240 mg/dL or was taking lipid-lowering agents. Current smoking was defined as having smoked within one year prior to being seen as an outpatient. A patient was considered to have CAOD if there was a history of acute myocardial infarction, unstable angina, angiographically confirmed CAOD, coronary artery bypass graft, or percutaneous coronary artery stent/angioplasty.

### Measurement of serum ALP and laboratory findings

The serum ALP level was measured in our central laboratory using a Hitachi 7600 automatic analyzer (HITACHI, Tokyo, Japan) that employed the p-nitrophenyl phosphate and diethanolamine method. The normal reference values of serum ALP range from 40 to 250 IU/L. Other laboratory data collected for analysis were as follows: white blood cell count, hematocrit, platelet count, total cholesterol, triglycerides, glucose, and estimated glomerular filtration rate (eGFR). The eGFR was calculated using the abbreviated Modification of Diet in Renal Disease Study Equation (186 × serum creatinine^−1.154^ × age^−0.203^ × 0.742 [*if female*)]) [14].

### Measurement of silent lacunar infarct (SLI), cWMH, and LCAS

Brain MRI and MRA were performed using one of three 1.5T MR systems (Sonata, Siemens Healthcare, Germany; Signa Excite, GE Healthcare, USA; Signa HDx, GE Healthcare, USA). Image interpretation was performed by one neurologist (H.B.L) and one radiologist (S.H.K), who was blinded with regard to clinical and laboratory data. An SLI was defined as a small (3–15 mm in diameter) cavitated lesion in an area supplied by deep perforating arteries, that showed high signal intensity on T1-weighted imaging (repetition time (TR)/echo time (TE) = 560/14 ms) and low signal intensity with a hyperintense rim on fluid-attenuation inversion recovery (FLAIR) imaging (TR/TE = 9000/105 ms, inversion time, 2500 ms) [15]. To evaluate the severity of cWMH, the Fazekas scale was used to quantify the periventricular and deep subcortical white matter on MRI FLAIR images [16, 17]. The Fazekas scores of the periventricular and deep subcortical white matter were added together, and the extent of severity of cWMH was defined as follows: no cWMH (total Fazekas score = 0), mild cWMH (total score = 1 or 2), moderate cWMH (total score = 3 or 4), and severe cWMH (total score = 5 or 6). The interobserver reliability of cWMH scoring was acceptable (Kappa coefficient = 0.63).

LCAS was identified as either marked stenosis (≥ 50%) or total occlusion of an intracranial or extracranial cerebral artery on brain MRA (MAGNETOM Symphony, Siemens, Heidelberg, Germany) [18]. Intracranial artery stenosis (ICAS) was identified in the following arteries: anterior cerebral, middle cerebral, posterior cerebral, distal internal carotid, distal vertebral and basilar arteries, using the method described in the Warfarin versus Aspirin for Symptomatic Intracranial Disease study [19]. Extracranial artery stenosis (ECAS) was identified in the proximal internal carotid and vertebral arteries, using the method described in the North American Symptomatic Carotid Endarterectomy Trial study [20].

### Statistical analysis

To evaluate the factors associated with serum ALP level, the participants were grouped into tertiles based on serum ALP levels (first tertile ALP ≤ 155 IU/L, second tertile 156–194 IU/L, third tertile ≥195 IU/L). Between-group assessments used analysis of variance for continuous variables and the Chi-square test for categorical variables. For purposes of some of the cWMH assessments, patients were divided into 2 groups based on the following severity scores: no moderate-to-severe (MS)-cWMH (Fazekas score = 0–2) and MS-cWMH (Fazekas score = 3–6). Univariate and multivariate logistic regression analyses were performed to evaluate the factors associated with SLI or cWMH. The logistic models, odds ratios (OR), and 95% confidence intervals (95% CIs) were determined using SLI or MS-cWMH as dependent variables. Adjustments were performed for the following established vascular risk factors: age, gender, hypertension, diabetes, hyperlipidemia, CAOD, and current smoking. To better understand the effect of ALP levels, we drew spline plots of the estimated probability of the occurrence of SLI and MS-cWMH according to ALP levels, using the generalized additive model. All statistical analyses were performed using the R package for Windows (version 3.1.3; R Foundation for Statistical Computing, Vienna, Austria). A two-sided *p* < 0.05 was considered statistically significant.

## Results

The mean age of the 1,011 study participants was 64.2 ± 9.1 years, and 64.5% were women. The mean serum ALP level was 183.06 ± 57.98 IU/L. The prevalences of SLI and MS-cWMH were 11.9% and 29.1%, respectively. The prevalences of LCAS, ICAS, and ECAS were 19.1%, 10.0%, and 11.7%, respectively. Analysis of the clinical characteristics of the participants divided according to ALP tertile found that the third tertile was significantly associated with female gender, higher WBC and platelet counts, higher triglyceride levels, SLI, and MS-cWMH, compared with the first tertile (Table 1). There were no differences in the prevalences of LCAS, ICAS, and ECAS across serum ALP tertiles.

**Table 1:** Demographic and laboratory data of 1,011 study participants according to serum ALP tertile.

	1^st^ tertile(≤ 155 IU/L) (n = 342)	2^nd^ tertile(156–194 IU/L) (n = 333)	3^rd^ tertile(≥ 195 IU/L)(n = 336)	*p*
Age (years)	63.5 ± 9.6	64.0 ± 9.0	65.0 ± 8.6	0.074
Gender (Female, %)	205 (59.9)	209 (62.8)	238 (70.8)*	0.009
Hypertension (%)	187(54.7)	185 (55.6)	207 (61.6)	0.141
Diabetes mellitus (%)	76 (22.2)	66 (19.8)	82 (24.4)	0.361
Hyperlipidemia (%)	96 (28.1)	116 (34.8)	120 (35.7)	0.068
CAOD (%)	20 (5.8)	16 (4.8)	16 (4.8)	0.769
Smoking (%)	74 (21.6)	67 (20.1)	64 (19.0)	0.701
Statin medication (%)	67 (19.6)	78 (23.4)	82 (24.4)	0.283
SBP (mmHg)	130.6 ± 18.3	131.5 ± 17.0	133.0 ± 19.6	0.235
DBP (mmHg)	79.7 ± 12.2	80.0 ± 10.8	80.4 ± 11.6	0.761
diff-BP (mmHg)	50.9 ± 14.1	51.4 ± 13.2	52.6 ± 14.5	0.267
WBC (× 10^9^/L)	6.3 ± 1.9	6.7 ± 2.0*	6.7 ± 1.9*	0.010
Hematocrit (%)	39.9 ± 4.0	40.0 ± 4.6	40.1 ± 3.8	0.820
Platelet (× 10^9^/L)	226 ± 52	235 ± 64	239 ± 60*	0.012
eGFR (mL/min/1.73m^2^)	74.4 ± 17.3	73.8 ± 16.9	74.1 ± 16.3	0.926
Uric acid (mg/dL)	4.55 ± 1.38	4.55 ± 1.41	4.47 ± 1.39	0.645
GOT (IU/L)	22.8 ± 7.7	23.4 ± 9.0	24.6 ± 11.6	0.056
GPT (IU/L)	22.5 ± 11.6	23.2 ± 12.1	25.4 ± 27.5	0.105
Total cholesterol (mg/dL)	191.2 ± 38.1	194.5 ± 38.3	195.8 ± 42.9	0.303
Triglyceride (mg/dL)	137.6 ± 82.2	151.8 ± 100.6	159.4 ± 100.7*	0.010
Fasting glucose (mg/dL)	126.8 ± 47.6	126.1 ± 45.1	131.2 ± 52.0	0.324
SLI (%)	29 (8.5)	36 (10.8)	55 (16.4)*	0.005
MS-cWMH (%)	86 (25.1)	89 (26.7)	119 (35.4)*	0.007
LCAS (%)	60 (17.5)	67 (20.1)	66 (19.6)	0.662
ICAS (%)	30 (8.8)	36 (10.8)	35 (10.4)	0.643
ECAS (%)	41 (12.0)	39 (11.7)	38 (11.3)	0.962

* Statistically significant compared to 1^st^ tertile by post-hoc analysis

ALP: Alkaline phosphatase; CAOD: coronary artery occlusive disease; SBP: systolic blood pressure; DBP: diastolic blood pressure; diff-BP: difference between SBP and DBP. WBC: white blood cells, eGFR: estimated glomerular filtration rate; GOT: glutamic oxaloacetic transaminase; GPT: glutamic pyruvic transaminase; SLI: silent lacunar infarct; MS-cWMH: moderate-to-severe cerebral white matter hyperintensities; LCAS: large cerebral arterial stenosis; ECAS: extracranial arterial stenosis; ICAS: intracranial arterial stenosis.

After adjustment for cardiovascular risk factors, multivariate analysis found that the patients in the third ALP tertile were more likely to have SLI (OR: 2.09; 95% CI: 1.27–3.42; *p* = 0.004) or MS-cWMH (OR: 1.48; 95% CI; 1.03–2.13, *p* = 0.036) compared with patients in the first tertile (Table 2). Further adjustment for eGFR, WBC count, triglyceride level, platelet count, and LCAS in this multivariate model did not change the association between the third tertile and presence of SLI (OR: 2.00; 95% CI: 1.20–3.31; *p* = 0.007) and MS-cWMH (OR: 1.46; 95% CI: 1.01–2.12; *p* = 0.044).

**Table 2:** Logistic regression analysis of the presence of SLI, MS-cWMH, LCAS, ICAS, and ECAS in 1,011 Korean participants according to serum ALP tertile.

	SLI		MS-cWMH		LCAS		ICAS		ECAS	
	OR (95% CI)	*p*	OR (95% CI)	*p*	OR (95% CI)	*p*	OR (95% CI)	*p*	OR (95% CI)	*p*
Unadjusted										
1^st^ tertile (≤ 155 IU/L)	Ref		Ref		Ref		Ref		Ref	
2^nd^ tertile (156–194 IU/L)	1.31 (0.78–2.19)	0.306	1.09 (0.77–1.53)	0.640	1.18 (0.80–1.74)	0.392	1.26 (0.76–2.10)	0.373	0.97 (0.61–1.55)	0.912
3^rd^ tertile (≥ 195 IU/L)	2.11 (1.31–3.41)	0.002	1.63 (1.17–2.27)	0.004	1.15 (0.78–1.69)	0.483	1.21 (0.72–2.02)	0.468	0.94 (0.59–1.50)	0.783
Adjusted*										
1^st^ tertile (≤ 155 IU/L)	Ref		Ref		Ref		Ref		Ref	
2^nd^ tertile (156–194 IU/L)	1.34 (0.79–2.26)	0.280	1.03 (0.71–1.51)	0.870	1.21 (0.81–1.82)	0.352	1.29 (0.76–2.19)	0.340	0.97 (0.60–1.56)	0.894
3^rd^ tertile (≥ 195 IU/L)	2.09 (1.27–3.42)	0.004	1.48 (1.03–2.13)	0.036	1.13 (0.75–1.69)	0.568	1.11 (0.66–1.89)	0.694	0.92 (0.57–1.51)	0.751

*Adjusted for age, gender, hypertension, diabetes, hyperlipidemia, coronary artery occlusive disease, and smoking.

Ref, reference group for statistical analysis; ALP: Alkaline phosphatase; SLI, silent lacunar infarct; MS-cWMH, moderate-to-severe cerebral white matter hyperintensities; LCAS, large cerebral arterial stenosis; ICAS, intracranial arterial stenosis; ECAS, extracranial arterial stenosis; OR, odds ratio; CI: confidence interval.

**Fig 1:**
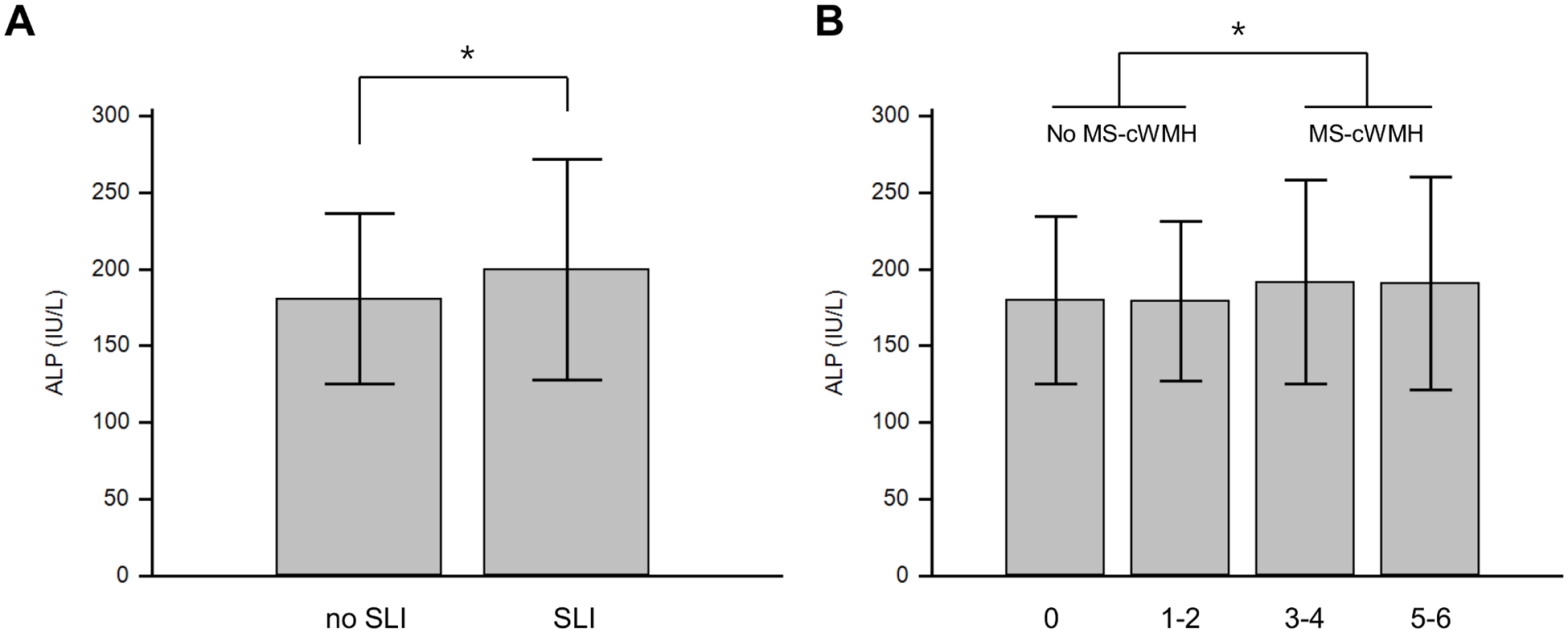
Mean concentration of serum ALP in participants with SLI and MS-cWMH. (A) Serum ALP levels between SLI and no SLI groups. (B) Serum ALP levels between four cWMH groups according to Fazekas score. Error bar indicates standard deviation. * *p* < 0.05. ALP: Alkaline phosphatase; SLI, silent lacunar infarct; MS-cWMH, moderate-to-severe cerebral white matter hyperintensities.

**Fig 2:**
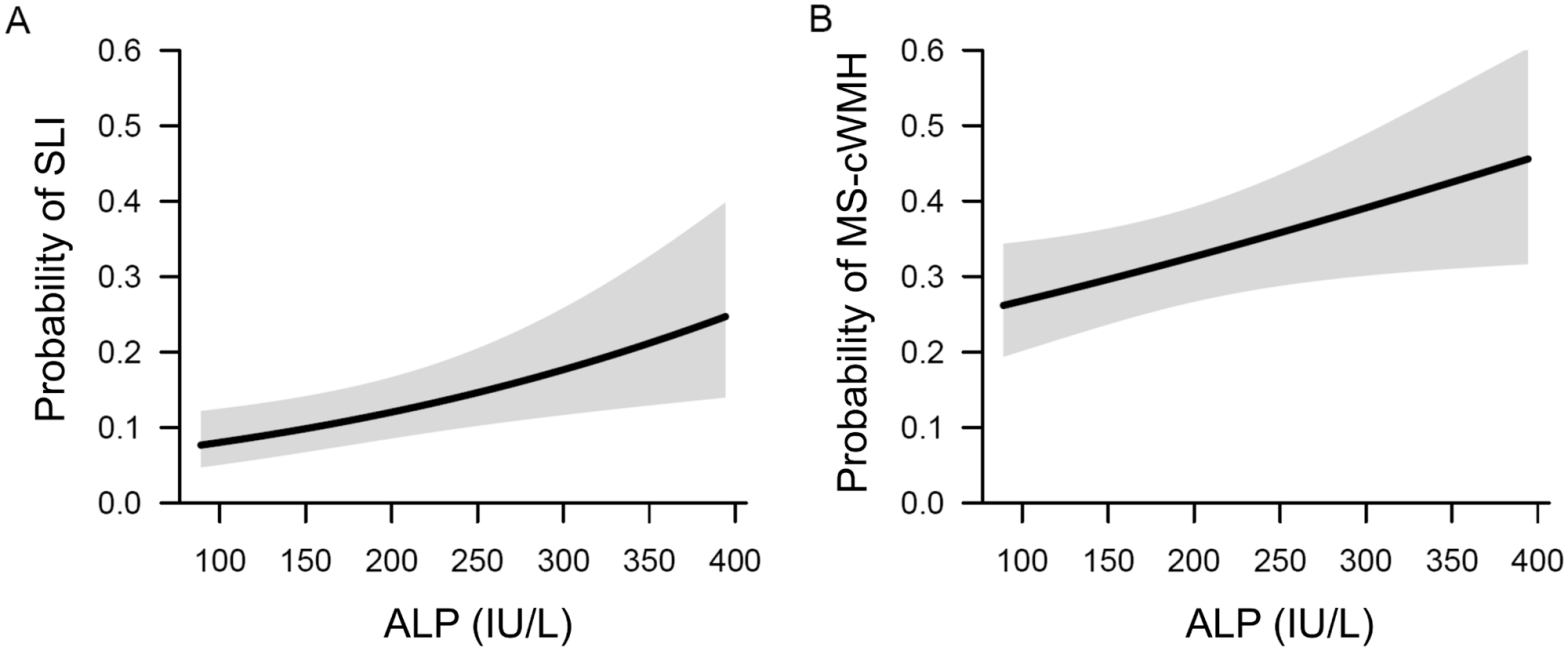
Spline curves of the relationship between ALP level and the presence of SLI and MS-cWMH†. (A) Relationship between ALP level and SLI. (B) Relationship between ALP level and MS-cWMH. The black lines and gray shadows represent the estimated probability and 95% confidence intervals for the presence of SLI and MS-cWMH, and were drawn using the generalized additive model. The x-axis is limited from the 1^th^ to 99^th^ percentile of ALP. †Adjusted for age, gender, hypertension, diabetes, hyperlipidemia, coronary artery occlusive disease, and smoking. ALP: Alkaline phosphatase; SLI, silent lacunar infarct; MS-cWMH, moderate-to-severe cerebral white matter hyperintensities.

The mean serum ALP level was significantly higher in patients with SLI (SLI *vs*. no SLI, 199.6 ± 71.8 IU/L *vs*. 180.8 ± 55.5 IU/L, *p* = 0.007) and in those with MS-cWMH (MS-cWMH *vs*. no MS-cWMH, 191.8 ± 67.0 IU/L *vs*. 179.5 ± 53.5 IU/L, *p* = 0.005) (Fig 1). With adjustments for confounding factors, multivariate analysis found that the serum ALP level was statistically significant for SLI (OR: 1.05 per 10 IU/L increase in ALP; 95% CI: 1.02–1.08; *p* = 0.003) and MS-cWMH (OR: 1.03 per 10 IU/L increase in ALP; 95% CI: 1.00–1.06; *p* = 0.025) (Table 3). A spline curve created using a generalized additive model showed a significant positive association between ALP level and the probability of SLI and MS-cWMH (Fig 2).

**Table 3:** Logistic regression analysis of serum ALP in the presence of SLI and MS-cWMH.

	SLI		MS-cWMH		LCAS		ICAS		ECAS	
	OR (95% CI)*	*p*	OR (95% CI)	*p*	OR (95% CI)	*p*	OR (95% CI)	*p*	OR (95% CI)	*p*
Unadjusted	1.05 (1.02–1.08)	0.001	1.04 (1.01–1.06)	0.003	1.01 (0.98–1.03)	0.595	1.02 (0.98–1.05)	0.381	0.99 (0.95–1.02)	0.398
Adjusted^†^	1.05 (1.02–1.08)	0.003	1.03 (1.00–1.06)	0.025	1.01 (0.98–1.04)	0.670	1.01 (0.98–1.05)	0.480	0.99 (0.95–1.02)	0.484

* Odds ratio per 10 IU/L increase in serum ALP.

^†^ Adjusted for age, gender, hypertension, diabetes, hyperlipidemia, coronary artery occlusive disease, and smoking.

ALP: Alkaline phosphatase; SLI, silent lacunar infarct; MS-cWMH, moderate-to-severe cerebral white matter hyperintensities; LCAS, large cerebral arterial stenosis; ICAS, intracranial arterial stenosis; ECAS, extracranial arterial stenosis; OR, odds ratio; CI, confidence interval.

Additional adjustments for hematocrit and platelet count did not change the significance of ALP levels in the multivariate models. Together with increased ALP levels, significant determinants for SLI were old age (adjusted OR: 1.05; 95% CI: 1.03–1.08), male gender (adjusted OR: 1.67; 95% CI: 1.04–2.70), and hypertension (adjusted OR: 3.16; 95% CI: 1.90–5.26). Together with increased ALP levels, significant determinants for MS-cWMH were old age (adjusted OR: 1.05; 95% CI: 1.03–1.08) and hypertension (adjusted OR: 1.05; 95% CI: 1.02–1.08). The relationship between ALP level and LCAS (OR: 1.01; 95% CI: 0.98–1.04), or ICAS (OR: 1.01; 95% CI: 0.98–1.05), or ECAS (OR: 0.99; 95% CI: 0.95–1.02) was not significant.

## Discussion

In this study, we found a positive association between serum ALP and MR indices of cSVD. By contrast, the serum ALP level was not associated with the MR indices of LCAS. The strength of our study was the use of multiple MR indices for cSVD and LCAS, which allowed us to determine the impact of ALP on two distinct types of cerebrovascular diseases.

The major finding of our study is that serum ALP is independently associated with 2 indices of cSVD. Participants in the third ALP tertile had 2.1-fold and 1.5-fold increased risk for SLI and cWMH, respectively, compared to those in the first tertile. Our results strongly support the results of a previous study that found that high serum ALP was closely related to large-volume cWMH and high prevalence of silent brain infarction [13]. In that study, the volume of cWMH was 0.27 mL larger in participants with the highest ALP quartile than those with lowest ALP quartile [13]. In addition, the participants in the highest ALP quartile had 2.6-fold increased risk for SBI [13]. Based on that study and our findings, an elevated serum ALP level may be a marker for impaired cerebral microcirculation.

The mechanism for the association between ALP and cSVD is not fully understood. There is an established association between vascular calcification and ALP, and vascular calcification may contribute to cSVD by impairing the microcirculation. Vascular calcification of major cerebral arteries results in stiff vessel walls, which may reduce the microcirculation distal to the calcified arteries [21]. In support of this hypothesis, studies have found that arterial calcification of large arteries leads to increased prevalence of cSVD, including increased volume of cWMH and SBI [11, 12]. The Rotterdam study found that an increased volume of cWMH was associated with a high computed tomography (CT) calcium load of the intracranial arteries, and silent brain infarction was associated with a high CT calcium load of the extracranial arteries, suggesting that calcification in different vessel beds leads to different manifestations of cSVD [12]. Another study demonstrated that CT-assessed intracranial calcification was associated with increased volume of cWMH [22].

Recent studies have shown that pericytes may be involved in vascular calcification. Exposure to vascular stress induces pericytes to differentiate into osteoblast-like cells and secrete osteoprotegerin, leading ultimately to vascular calcification [23]. Because pericytes are abundant in the cerebral capillary system, cerebral white matter may be vulnerable to calcification of pericytes triggered by the aging process or vascular stress. Since ALP mediates vascular calcification by inducing collagen deposition, ALP-mediated venous collagenosis is also a candidate mechanism for the pathogenesis of cSVD [24].

Another possible reason accounting for the association between ALP and cSVD is that high serum ALP reflects inflammation, which is involved in the development of cSVD [2]. Van Dijk et al [25] found that patients with cSVD had high levels of C-reactive protein (CRP), which suggests a close relationship between vascular inflammation and cSVD. We did not evaluate CRP (or high-sensitivity CRP), and therefore the relationship between inflammation and cSVD could not be investigated in our study. However, a previous study showed that ALP and CRP additively increased the risk of cSVD [13], results suggesting that ALP and CRP are independent predictors of cSVD. Therefore, it is unlikely that an elevated serum ALP level is merely a marker of vascular inflammation in patients with cSVD.

Previous clinical studies have demonstrated that an elevated serum ALP level is associated with the risk of cardiovascular disease [2–5], suggesting that arterial calcification contributes to the development of cardiovascular events. However, we failed to find an association between serum ALP and cerebral LCAS, regardless of the location of stenosis. Our result are supported by a previous study showing that serum ALP was not associated with the presence or severity of ECAS and ICAS in 1,034 patients with ischemic stroke [6]. Although the reason for the lack of association between serum ALP and cerebral LCAS is unclear, different blood vessels (aorta, coronary artery, cerebral artery) have different susceptibility to the development of atherosclerosis [26].

In addition, ALP enhances medial calcification longitudinally along the vascular wall without focal narrowing [27], and this type of lesion may be undetectable on MRA. Thus, arterial calcification may serve as a pathological substrate for atherosclerotic disease, but may be not correlated directly with the severity of focal stenosis. In our study, we used a strict definition of LCAS (≥ 50% stenosis on brain MRA), and our findings may have underestimated the true impact of serum ALP on cerebral atherosclerosis, especially for the evaluation of intracranial arteries. In the clinical setting, the assessment of a mild degree of atherosclerosis in the intracranial arteries using brain MRA is difficult. Further studies that use conventional cerebral angiography and high-resolution intracranial MR imaging are needed for additional clarification.

Our study has several limitations. First, our study was retrospective and may have had a selection bias. Although a prospective study would be preferable, the recruitment of a sufficient number of participants who have undergone brain MRI and MRA is methodologically difficult at a single institution. Second, our study patients were neurologically healthy but sought medical attention for underlying vascular risk factors. These patients therefore had a higher prevalence of cardiovascular risk factors than would be found in a general healthy population. Third, we measured the total ALP activity. Since serum ALP activity is derived from various tissues of origin, an ALP bone isoenzyme assay might be better for clarifying whether there is an independent association between elevated ALP and cSVD. However, an ALP bone isoenzyme assay is not routinely performed in the clinical setting. Moreover, most of the association studies that have investigated serum ALP levels in vascular disease have measured the total ALP activity; therefore our results could be directly compared with the results of previous studies [2, 7]. Fourth, the mechanism that underlies the association between serum ALP and cSVD remains unclear. We could not conclude whether serum ALP is a risk factor or merely a marker for cSVD. Finally, although we collected and adjusted for as many risk factors and laboratory findings as possible, we had no data on liver disease (gamma glutamyl transferase levels) and bone disease (parathyroid hormone levels) from our cohort. Recent studies have suggested that migraine [28, 29] and osteoporosis [30, 31] may be independently associated with cSVD. Our study had no data on migraines or osteoporosis. Since there was a high proportion of women in our study, those factors might have affected the results. Further studies are needed to elucidate the association between cSVD and migraine and between cSVD and osteoporosis.

## Conclusions

Our study found a positive association between serum ALP levels and MR indices of cSVD, such as SLI and cWMH. On the other hand, serum ALP was not associated with LCAS, regardless of the location of stenosis. Although further studies are needed, our results suggest that elevated serum ALP levels contribute to the development of cSVD.

## Supporting Information

S1 File File containing patient information. (XLS)
